# Bacteriophage-based tools: recent advances and novel applications

**DOI:** 10.12688/f1000research.9705.1

**Published:** 2016-11-29

**Authors:** Lisa O'Sullivan, Colin Buttimer, Olivia McAuliffe, Declan Bolton, Aidan Coffey

**Affiliations:** 1 Department of Biological Sciences, Cork Institute of Technology, County Cork, Ireland; 2 Biotechnology Department, Teagasc, Moorepark Food Research Centre, Fermoy, County Cork, Ireland; 3 Division of Food Safety, Teagasc, Food Research Centre, Ashtown, County Dublin, Ireland

**Keywords:** bacteriophage, phage, phage research, biocontrol, bionanotechnology, bacterial diagnostics, phage-based drug delivery, biotechnology

## Abstract

Bacteriophages (phages) are viruses that infect bacterial hosts, and since their discovery over a century ago they have been primarily exploited to control bacterial populations and to serve as tools in molecular biology. In this commentary, we highlight recent diverse advances in the field of phage research, going beyond bacterial control using whole phage, to areas including biocontrol using phage-derived enzybiotics, diagnostics, drug discovery, novel drug delivery systems and bionanotechnology.

## Introduction

Bacteriophages (phages) are viruses that specifically infect bacteria. After their discovery in 1915 by Twort and 1917 by d’Herelle, these agents were initially used to treat bacterial infections, although widespread acceptance was limited owing to lack of understanding of phage biology and the development of antibiotic therapy in the 1940s ^1^. With antibiotic resistance becoming problematic in the late twentieth century ^2^, there was a renewed interest in phage therapy research. Alongside this application, and indeed the fundamental role that phage research played in the understanding of molecular biology, phage research has led to the development of new technologies not only for therapy and biocontrol but also for bacterial detection, drug delivery, drug discovery, and nanotechnology.

## Antibacterials and biocontrol

In addition to the well-documented cases of using wild-type phages as tools to eliminate pathogenic bacteria in infected humans ^3^ and in foods ^4^, the phage-encoded peptidoglycan hydrolases called endolysins have also been exploited in purified form to rapidly lyse bacterial cells ^5^. The Gram-positive phage endolysins generally contain at least one enzymatic domain and a cell-wall-binding domain. Chimeric endolysins have recently been developed by fusing enzymatic domains to alternative cell-wall-binding determinants, thus altering endolysin behaviour and host range ^6^. In the case of Gram-negative bacteria, the outer membrane is a barrier to exogenously added endolysin reaching the peptidoglycan target. Thus, the fusion of polycationic peptides to the Gram-negative endolysin facilitates outer membrane penetration allowing these new so-called Artilysin®s access to the Gram-negative peptidoglycan ^7^. Recent research has also reported a phage endolysin (from a *Streptococcus pyogenes* phage) with the ability to cross mammalian cell membranes. Its endolysin, PlyC, was found to consist of two subunits, one of which is proposed to bind to the eukaryotic cell membrane, facilitating entry by endocytosis ^8^. These are major breakthroughs in endolysin research, and, with further investigation and testing, similar enzymes may be discovered/engineered and used in the future to, respectively, treat infections caused by Gram-negative bacteria and intracellular bacterial infections.

A recent advance in the area of antibiotic therapy has been the exploitation of phages to control antibiotic-resistant bacteria. Phages have been engineered to deliver CRISPR-Cas nucleases into antibiotic-resistant bacterial cells, and, in doing so, researchers have been able to harness the specific DNA-cleaving capacity of CRISPRs to knock out antibiotic resistance sequences, rendering resistant organisms antibiotic sensitive ^9^. The use of phages as delivery vehicles ensures the specificity required in biocontrol. The wider exploitation of phages as delivery systems is discussed below.

## Bacterial diagnostics

Phage virions and their encoded proteins can also be useful for the detection and specific identification of bacteria. The simplest of these is where a standard number of specific phages are incubated with a food material or some other test sample. If the bacterial target is present and viable, detectable phage numbers will increase through amplification on the pathogen. Modifications of this method can generate results more rapidly, and in the case of *Yersinia pestis*, Sergueev *et al*., for example, developed a quantitative real-time PCR to detect the increase in phage DNA instead of traditional plaque assays ^10^. Reporter phages can also detect bacteria through infection without needing cell lysis and progeny phages. In this case, the phage genomes are modified to carry a bioluminescence or fluorescence gene that the phage alone cannot express. Upon injection of the phage DNA into its host, active bioluminescent or fluorescent proteins are synthesized, facilitating visual detection. Recently, Zhang *et al*. engineered an *Escherichia coli* 0157:H7 reporter phage containing Luciferase NanoLuc (Nluc) ^11^ and with it detected as few as five CFU of the *E. coli* by bioluminescence in a complex food matrix within nine hours ^12^.

Reporter phage assays have also been adapted to assess drug sensitivity in the target bacterium. A *Mycobacterium tuberculosis* (TB) fluorophage, ϕ ^2^GFP10, has been shown to detect TB in the complex matrix of a sputum sample, but also when rifampin or kanamycin are included in the assay, fluorescence was shown to be detectably diminished in sensitive cells in comparison with antibiotic-resistant variants ^13^. Advantages of using whole phages for the detection of bacteria are that only viable bacterial cells are detected, bacterial host specificity is excellent, and phage cultivation is relatively inexpensive (however, lytic activity of a reporter phage should ideally be inactivated to ensure that the bacterial targets are not prematurely destroyed).

Phage receptor-binding proteins (RBPs) have also been used successfully in bacterial detection and identification. The receptor-binding domain of the RBP in *Campylobacter* phage NCTC12673 was used to create a simple glass slide agglutination test for *Campylobacter*, and when fused to green fluorescent protein, the receptor-binding domain allowed the detection of *Campylobacter jejuni* and *Campylobacter coli* isolates through fluorescent microscopy ^14^. Phages, because of their vast diversity, provide a plentiful source of host-specific proteins to create simple identification tests such as the agglutination assay mentioned above specifically for *Campylobacter*. In this regard, whole phage and phage RBPs have been successfully attached to biosensing surfaces for bacterial detection, allowing for high specificity. Of the two, the RBPs are simpler and easier to attach. In addition, they can be recombinantly produced and are reported to have better stability than antibodies ^15^. Optimization of phage densities and attachment to biosensing surfaces is still ongoing ^16^.

In the context of detection, the phage endolysins (discussed earlier) can also have a role when used instead of traditional DNA extraction reagents. It was shown that the peptidoglycan of *Staphylococcus aureus* is degraded more rapidly by staphylococcal endolysin ClyH than by lysostaphin, thus shortening the DNA sample preparation for real-time PCR when the endolysin was employed ^17^. Phage display, which involves genetically modifying a phage virion so that a foreign peptide is displayed on the surface (discussed further below), can also be exploited in bacterial detection systems. Lee *et al*. created a phage that displayed two different peptides, one with an affinity to gold nanoparticles and another with specificity to a target protein. By measuring the ultraviolet absorbance of this phage, they could detect as little as 25 femtomoles of their target antigen ^18^. These modified phages have also been incorporated into systems capable of in-the-field real-time detection using engineered phage displaying peptides capable of binding to a magnetoelastic resonator as well as the target analytes, such as bacteria and endospores ^19– 21^.

## Drug discovery and phage-based drug delivery systems

Since phage display was first described in 1985 by Smith ^22^, it has seen numerous applications in the identification of receptor and ligand interactions of infectious diseases and cancers ^23, 24^, with these developments allowing for drug discovery ^25^ and vaccine design ^26^. Phage display is now allowing the modification of phages into vehicles (nanocarriers) for chemotherapeutic drug delivery by the attachment of a drug to the phage surface and presentation of peptides on the surface of that phage with specificity to a ligand of interest. Such constructs have even been designed to target non-host bacteria, including mammalian cells ^27^. These phages, displaying therapeutic peptides, can even be designed to pass the blood–brain barrier ^28^, and such constructs could thus have potential in the treatment of diseases such as Alzheimer’s and Parkinson’s. Phages with an affinity to specific cell receptors, such as those overexpressed in cancer cells, may be exploited beyond drug delivery to allow for simultaneous target detection by displaying diagnostic reporter molecules or by detection of bound phage DNA by real-time PCR ^29, 30^.

Empty phage capsids are also being employed as carriers, with studies demonstrating the potential to encapsulate RNA molecules, peptides, and therapeutic compounds ^31– 33^. Phage capsids or virus-like particles (VLPs) have also been modified to present ligands on their surface to allow the delivery of encapsulated RNA-guided endonucleases to specific cell types for *in situ* genome editing ^34^. When phages are used as nanocarriers to deliver chemotherapeutic drugs for cancer treatment, drug half-life is extended and toxicity is focused only on the site of interest, lessening damage to body tissues ^35^. Capsid-based carriers have also been developed by fusing drug-loaded liposomes to capsid proteins displaying peptides with binding specificity to a particular target ^36^.

## Biotechnology

Genetically modified filamentous phages have been used in material synthesis to construct nanowires and films for semi-conductor applications ^37^, piezoelectric energy generation ^38^, and photo-response properties ^39^. These materials have been used to create devices such as ion batteries and catalysts ^40, 41^, with phage M13-based nanowires also being constructed into scaffolding to allow guided cell growth for human tissue formation ^42^.

Phage-derived enzymes, which have formed part of a core toolbox in traditional molecular biology, are now being applied to novel concepts. Phage RNA polymerase and ribonuclease H are being used to create *in vitro* genetic circuits that have potential future applications in nanodevices and the regulation of processes within artificial cells ^43^. Recombinases are seeing use in these constructions by extending memory capabilities to these circuits ^44^. These enzymes are also being used to create novel tools for bacterial genome editing and accelerated evolution ^45, 46^. It is noteworthy that many past phage-dedicated reviews have not satisfactorily encompassed the recent advances of phage applications in nanomedicine; a recent excellent article comments in a comprehensive way on the many roles and opportunities of phages as nano-therapeutics, bioimaging probes, biomimetic biomaterials, tissue regenerative scaffolds, matrices for directing stem cell fate, and probes for detecting disease biomarkers, among numerous others ^47^.

## Summary

This commentary provides a snapshot of the increasing diversity of phage research in recent years and shows that it is advancing rapidly and that new applications are being reported frequently. Since the discovery of phages a century ago, their research focus has diversified from applying these agents to simply treat bacterial infections to a broad range of useful functions including biocontrol, diagnostics, drug discovery, and drug delivery as well as several applications in nanomedicine.
